# *Streptomyces albidoflavus* Q antifungal metabolites inhibit the ergosterol biosynthesis pathway and yeast growth in fluconazole-resistant *Candida glabrata*: phylogenomic and metabolomic analyses

**DOI:** 10.1128/spectrum.01271-23

**Published:** 2023-09-27

**Authors:** Celia Bautista-Crescencio, Arturo Casimiro-Ramos, M. Jonathan Fragoso-Vázquez, José Correa-Basurto, Carlos Olano, César Hernández-Rodríguez, Lourdes Villa-Tanaca

**Affiliations:** 1 Departamento de Microbiología, Laboratorio de Biología Molecular de Bacterias y Levaduras, Escuela Nacional de Ciencias Biológicas, Instituto Politécnico Nacional. Prolongación de Carpio y Plan de Ayala, Casco de Santo Tomás, Ciudad de México, Ciudad de México, México; 2 Departamento de Química Orgánica, Escuela Nacional de Ciencias, Biológicas, Instituto Politécnico Nacional, Prolongación de Carpio y Plan de Ayala, Col. Casco de Santo Tomás, Ciudad de México, México; 3 Laboratorio de Diseño y Desarrollo de Nuevos Fármacos e Innovación Biotecnológica (Laboratory for the Design and Development of New Drugs and Biotechnological Innovation), SEPI-Escuela Superior de Medicina del Instituto Politécnico Nacional, Plan de San Luis y Salvador Díaz Mirón, Casco de Santo Tomás, Ciudad de México, México; 4 Departamento de Biología Funcional, Universidad de Oviedo, Oviedo, Spain; Agricultural Research Organization Volcani Center, Rishon LeZion, Israel

**Keywords:** antifungal, multi-drug resistance, *Candida glabrata*, *Streptomyces albidoflavus*, WGS, metabolomics, ergosterol, HMGR (EC 1.1.1.34), cytoplasmic membrane, actinomycete, actinobacteria, plant-associated metabolites

## Abstract

There is an urgent need to develop new antifungals due to the increasing prevalence of multidrug-resistant fungal infections and the recent emergence of COVID-19-associated candidiasis. A good study model for evaluating new antifungal compounds is *Candida glabrata*, an opportunistic fungal pathogen with intrinsic resistance to azoles (the most common clinical drugs for treating fungal infections). The aim of the current contribution was to conduct *in vitro* tests of antifungal metabolites produced by the bacteria *Streptomyces albidoflavus* Q, identify their molecular structures, and utilize several techniques to provide evidence of their therapeutic target. *S. albidoflavus* was isolated from maize rhizospheric soil in Mexico and identified by phylogenomic analysis using a 92-gene core. Of the 66 metabolites identified in *S. albidoflavus* Q by a liquid chromatography-high resolution mass spectrometry (LC-HRMS) metabolomic analysis of the lyophilized supernatant, six were selected by the Way2drug server based on their *in silico* binding to the likely target, 3-hydroxy-3-methyl-glutaryl-CoA reductase (HMGR, the key enzyme in the ergosterol biosynthesis pathway). Molecular modeling studies show a relatively high binding affinity for the *Cg*HMGR enzyme by two secondary metabolites: isogingerenone B (diaryl heptanoid) and notoginsenoside J (polycyclic triterpene). These secondary metabolites were able to inhibit ergosterol synthesis and affect yeast viability *in vitro*. They also caused alterations in the ultrastructure of the yeast cytoplasmic membrane, as evidenced by transmission electron microscopy. The putative target of isogingerenone B and notoginsenoside J is distinct from that of azole drugs (the most common clinical antifungals). The target for the latter is the lanosterol 14 alpha-demethylase enzyme (Erg11).

**IMPORTANCE:**

Multidrug resistance has emerged among yeasts of the genus *Candida*, posing a severe threat to global health. The problem has been exacerbated by the pandemic associated with COVID-19, during which resistant strains of *Candida auris* and *Candida glabrata* have been isolated from patients infected with the SARS-CoV-2 virus. To confront this challenge, the World Health Organization has invoked scientists to search for new antifungals with alternative molecular targets. This study identified 66 metabolites produced by the bacteria *Streptomyces albidoflavus* Q, 6 of which had promising properties for potential antifungal activity. The metabolites were tested *in vitro* as inhibitors of ergosterol synthesis and *C. glabrata* growth, with positive results. They were also found to damage the cytoplasmic membrane of the fungus. The corresponding molecular structures and their probable therapeutic target were established. The target is apparently distinct from that of azole drugs.

## INTRODUCTION

Many fungal infections are difficult to treat, such as those caused by yeasts of the genus *Candida* spp. when associated with coronavirus disease 2019 (COVID-19), human immunodeficiency virus (HIV) (1) (https://www.cdc.gov/fungal/covid-fungal.html), or certain other infections. The treatment of difficult fungal infections is further complicated by the growing prevalence of multidrug resistance among *Candida* species, rendering the common clinical drugs (e.g., amphotericin B, azoles, and echinocandins) ineffective and creating a severe threat to global health (2). To respond to this crisis, a multidisciplinary approach is needed that includes the search for sources of new antifungals with alternative molecular targets, the rational design and development of new drugs and vaccines, and accurate and timely diagnoses (https://www.who.int/news-room/fact-sheets/detail/antimicrobial-resistance).

Among the *Candida* infections, which have become more frequent in recent years, *C. albicans* is the predominant cause of invasive infections (3). Invasiveness can also characterize healthcare-associated infections by *C. glabrata*, *C. parapsilosis*, *C. krusei*, and recently the multidrug-resistant *C. auris* (3). *C. glabrata* is a pathogenic yeast intrinsically resistant to azoles, and its recent pan-echinocandin-resistant clinical isolates are associated with the COVID-19 pandemic (2, 3). For this reason, it has become a model used in research to test new antifungal compounds for their effectiveness against pathogenic *Candida* species.

Bacteria of the Actinomycetes class, especially of the genus *Streptomyces*, are a possible source of new antifungals with alternative molecular targets. They produce numerous secondary metabolites with antifungal, antibacterial, and antiviral activities, including alkaloids, pigments, and toxins (4, 5). These metabolites are encoded by groups of genes denominated biosynthetic gene clusters (BGCs). In *Streptomyces*, antimicrobial metabolites are encoded by hundreds of BGCs, including those classified as cryptic, silent, and dormant (6 – 9). Methods have been found to activate the biosynthesis of such metabolites (10 – 12). Indeed, the study of antifungals produced by actinobacteria of the *Streptomyces* genus over the last several years (13) has led to the successful identification, elaboration, and application of amphotericin B, natamycin, and nystatin (Table 1).

**TABLE 1:** Examples of antifungal compounds generated by actinobacteria of the genus *Streptomy*ces spp., including commercial compounds and those in the developmental phase^*a*^

Strain of *Streptomyces* spp.	Antifungal compound	Reference
*Streptomyces noursei*	Nystatin (polyene) (commercial)	Campoy and Adrio (13). Doi: 10.1016/j.bcp.2016.11.019
*Streptomyces nodosus*	Amphotericin (polyene) (commercial)	Campoy and Adrio (13). Doi: 10.1016/j.bcp.2016.11.019
*Streptomyces natalensis*	Natamycin (polyene) (commercial)	Campoy and Adrio (13). Doi: 10.1016/j.bcp.2016.11.019
*Streptomyces rimosus*	Unknown	Lu et al. (14). Doi: 10.1002/jobm.201500666
*Streptomyces mutabilis*	2,4-Di-tert-butilfenol	Belghit et al. (15). Doi: 10.1016/j.mycmed.2016.03.001
*Streptomyces* spp. ERI-04	Unknown	Valanarasu et al. (16). Doi: 10.1016/j.mycmed.2010.09.001
*Streptomyces* spp. MTCC 5680	Macrolide polyene (PN00053)	Vartak et al. (17). Doi: 10.1111/lam.12229
*Streptomyces* spp. SNM55	Mohangamides A	Bae et al. (18). Doi: 10.1021/ol5037248
*Streptomyces albus* J1074	Surogamides	Xu et al. (19). Doi: 10.1021/jacs.7b02716
	Acyl-surugamides	
	Albucyclones	
	Albuquinone	

^*a*^ All the information was taken from the original reference article.

**Fig 1:**
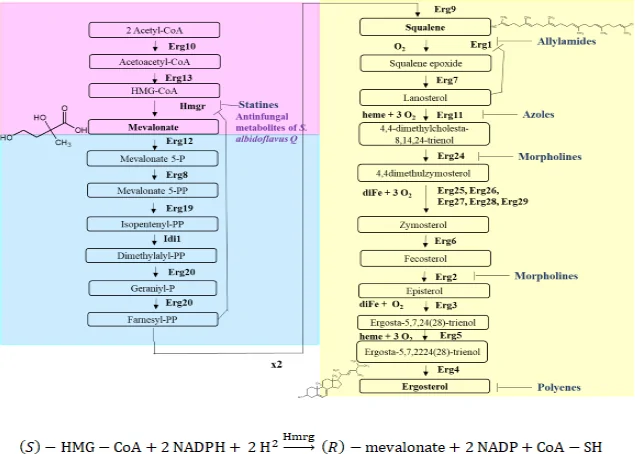
The biosynthetic pathway of ergosterol in yeasts, divided into three modules: the mevalonate pathway (in pink), the farnesyl pyrophosphate pathway (in blue), and the last step leading to ergosterol (in yellow). Enzymes, intermediates, inhibitors, and the requirements for oxygen, heme, and iron are indicated. Inhibitors are listed with their respective target: statins bind with HMGR, allylamines with Erg1, azoles with Erg11, morpholines with Erg2 and Erg24, and polyenes with ergosterol. 3-Hydroxy-3-methyl-glutaryl-CoA reductase (HMGR) is the proposed target for secondary metabolites produced by *Streptomyces albidoflavus*. Below the boxes, the reaction described is catalyzed by the HMGR enzyme with NADPH as a cofactor.

*Cg*HMGR has been proposed as an alternative therapeutic target for new antifungals (20 – 23). It participates in the mevalonate biosynthesis pathway, the first module of the ergosterol biosynthesis pathway (Fig. 1) (24). Since ergosterol plays a critical role in the homeostasis and function of the cytoplasmic membrane, the enzymes involved in its biosynthetic pathway are promising targets for the design of new antifungals (21, 25) (Fig. 1).

Besides the antifungals from *Streptomyces* already in clinical use (e.g., amphotericin B, nystatin, and natamycin), the existence of other possibly useful antifungal compounds has been evidenced as well. However, many reports on such metabolites have left various factors undefined, including the strain and species of *Streptomyces* from which they are derived, the chemical structure of the active compound, and the therapeutic target (Table 1). In the current contribution, the *Streptomyces* strain isolated from rhizospheric soil of Mexican maize was identified as *S. albidoflavus* Q by phylogenomic analysis. Possible antifungal metabolites were discovered by metabolomic analysis. Plausible biosynthetic pathways involved in their synthesis are suggested. The *Cg*HMGR enzyme is proposed as the likely therapeutic target of the metabolites tested *in vitro* based on their inhibition of ergosterol synthesis and experiments with growth recovery. These metabolites caused alterations in the cytoplasmic membrane, reduced the levels of ergosterol, and decreased the viability of *C. glabrata*. The probable mechanism of interaction between the antifungal metabolites and *Cg*HMGR was defined by molecular modeling of the ligands and a thorough examination of the alterations generated in the yeast cytoplasmic membrane.

## RESULTS

### Phenotypic characteristics of *Streptomyces albidoflavus* Q

**Fig 2:**
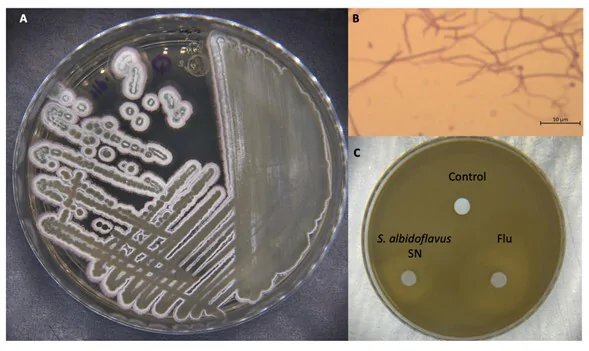
Phenotypic characteristics of *Streptomyces albidoflavus* Q. The strain was inoculated onto solid GAE medium and incubated at 28°C for 15 days. (**A**) Its colonial morphology is characteristic of actinobacteria. (**B**) The microscopic morphology corresponds to a Gram-positive bacteria with filamentous structures (1,000×). (**C**) *S. albidoflavus* metabolites inhibited *C. glabrata* growth. The supernatant (SN) of *S. albidoflavus* Q was concentrated by lyophilization and resuspended. Fluconazole (Flu) and water served as the positive and negative controls, respectively, for the inhibition of yeast growth (the modified M44 CLSI method).

First, the phenotype of the bacterial strain was identified. The colonial morphology was characteristic of actinobacteria (i.e., powdery-looking dry colonies), while microscopic morphology evidenced a Gram-positive bacteria with filamentous structures (Fig. 2A and B). The *S. albidoflavus* Q supernatant, which was obtained, lyophilized, and resuspended in sterile water (see Materials and Methods), exhibited inhibition of the confluent growth of fluconazole-resistant *C. glabrata* (Fig. 2C).

### Whole-genome sequencing of *Streptomyces albidoflavus* Q and phylogenomic analysis

**Fig 3:**
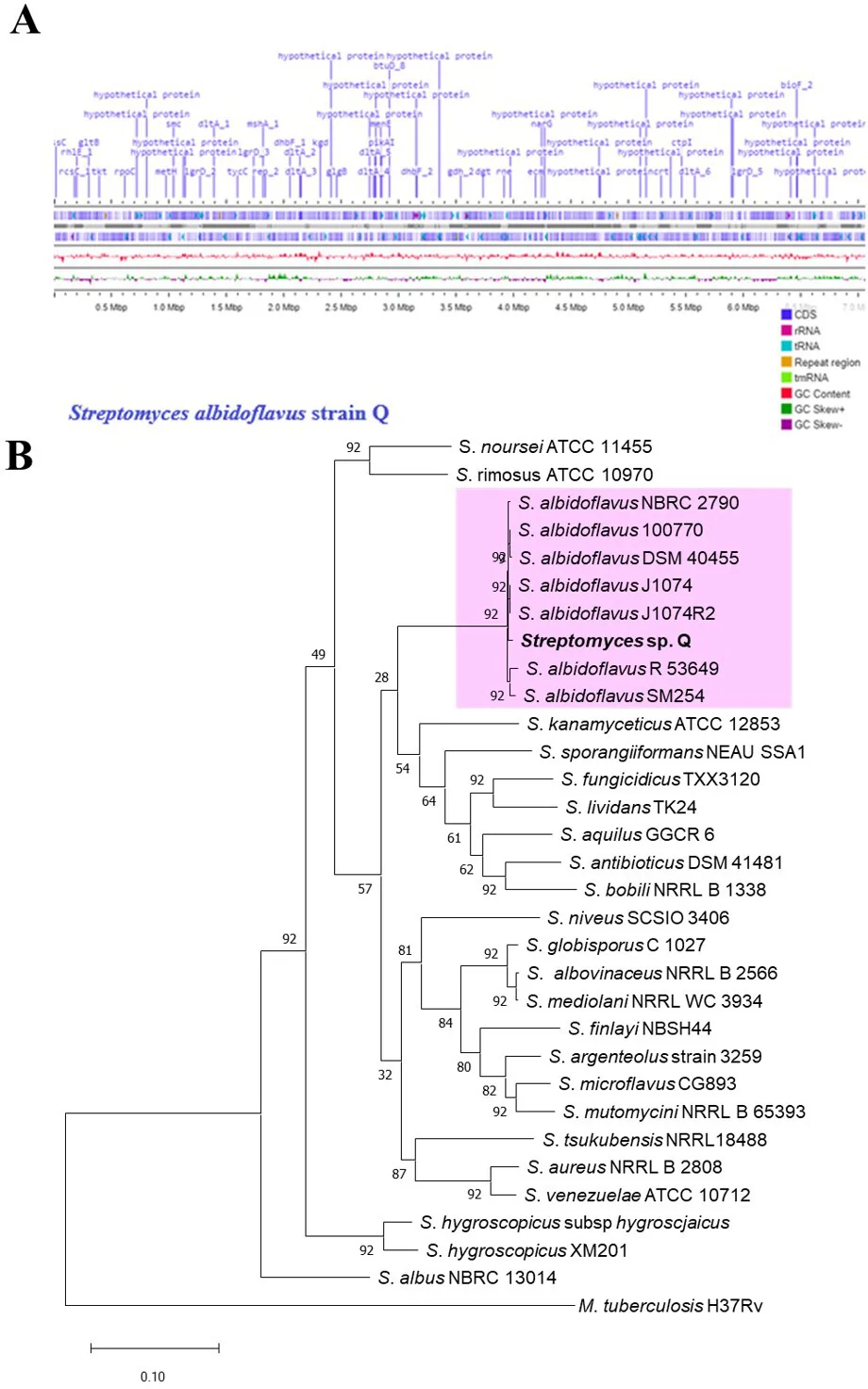
General features of the *Streptomyces albidoflavus* Q genome and the phylogenetic tree. (**A**) A linear representation of *S. albidoflavus* Q genome contigs was obtained with Proksee (https://proksee.ca). The scale is expressed in megabases (Mbp), and two dark blue lines denote forward and reverse strand CDSs, respectively. Some genes are portrayed in violet with the default setting of Proksee. The tRNA (blue arrows), rRNA (magenta arrows), and tmRNA (green arrows) are shown with violet lines. The content (in pink) and skew (in dark green and violet) of gene clusters are illustrated. (**B**) The phylogenomic tree of *S. albidoflavus* Q was inferred by concatenated alignment of 92 core genes (UBCGs). Gene support indices (GSIs) and percentage bootstrap values are given at branching points. Bars represent 0.10 substitution per position.

The quality control of the assembly estimated the total length of the contigs to be 6.95 Mbp for *S. albidoflavus* Q, with a G+C content of approximately 73% and an N50 of 425,381. There were 6,007 CDS, 6 rRNA, 96 tRNA, and 1 tmRNA (Fig. 3A).

The species of this actinobacteria was identified through a phylogenomic analysis (see Materials and Methods). The phylogenomic tree was constructed with the up-to-date bacterial core gene (UBCG) software, which uses 92 concatenated genes to generate a maximum likelihood tree with gene support index values (Fig. 3B). Sequences of *Streptomyces* spp. and *S. albidoflavus* strains from other ecosystems, such as rhizosphere soil, marine soil, and the gut of an ant, are stored in the NCBI server.

### Antifungal activity of the lyophilized supernatant of *Streptomyces albidoflavus* Q on *Candida glabrata*

**Fig 4:**
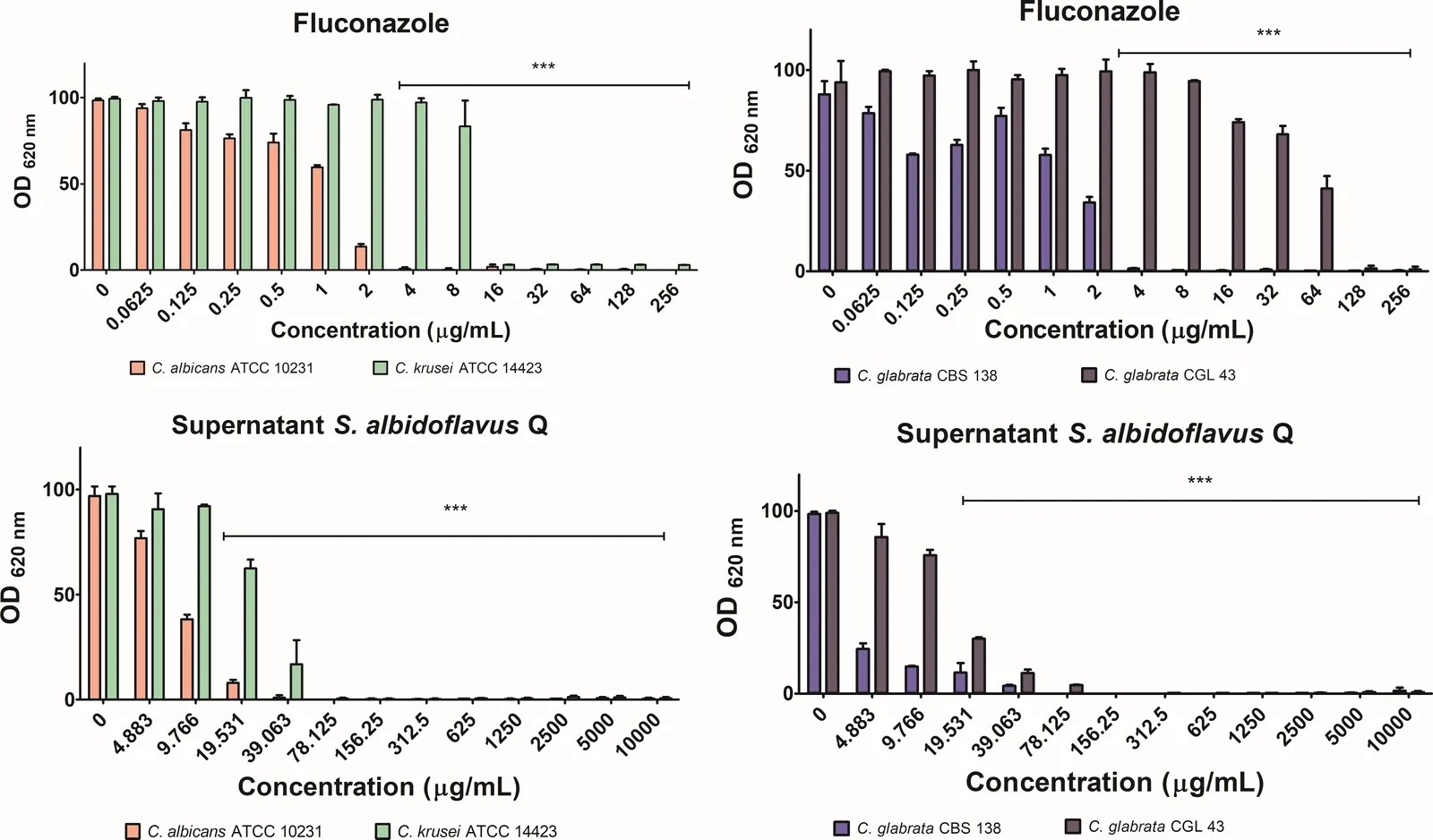
Inhibition of the growth of *Candida* spp. While growth without any inhibitor served as the negative control, fluconazole was used as the positive control of inhibitory activity. The optical density was determined in a Thermo Scientific Multiskan FC microplate photometer at 620 nm (OD_620_) after incubation at 37°C for 24 h. The quantification of growth was expressed as the average ± standard deviation (SD) of optimal density values from three independent assays. Significant differences were analyzed by two-way ANOVA. ***P < 0.001.

*C. glabrata* CGL 43 was adopted as the study model due to its intrinsic resistance to fluconazole. The controls consisted of two strains susceptible to the same antifungal drug: another strain of *C. glabrata* (CBS 138) and a *C. albicans* strain (ATCC 10231). *C. krusei*, which like *C. glabrata* CGL 43 is resistant to fluconazole, also served as a control (Fig. 4).

Although fluconazole acts on the ergosterol biosynthesis pathway, its therapeutic target is not HMGR, but rather the enzyme encoded by *ERG11* (lanosterol 14 alpha-demethylase). This enzyme participates in one of the last steps of ergosterol synthesis (Fig. 1). Fluconazole was used as a positive control.

For the evaluation of inhibition, the lyophilized supernatant of *S. albidoflavus* Q was applied at different concentrations. As the concentration increased, the growth of the yeast strain decreased, indicating a concentration-response effect (Fig. 4). A determination was made of the minimum inhibitory concentration that inhibits the growth of *Candida* spp. by 50% (MIC_50_) and 70–90% (MIC_70-90_). *S. albidoflavus* Q metabolites were able to inhibit the growth of *C. glabrata* CGL 43. The MIC_50_ and MIC_70-90_ values were lower than those of fluconazole (Table 2).

**TABLE 2:** MIC_50_ and MIC_70-90_ values of fluconazole and the lyophilized supernatant of *S. albidoflavus* Q in relation to four *Candida* spp.

	*C. albicans* ATCC 10231		*C. krusei* ATCC 14423		*C. glabrata* CBS 138		*C. glabrata* CGL 43	
Inhibitor	MIC_50_	MIC_70–90_	MIC_50_	MIC_70–90_ (µg/mL)	MIC_50_	MIC_70–90_	MIC_50_	MIC_70-90_
Control^*a*^	–	–	–	–	–	–	–	–
Fluconazole	1.25	2	12	16	1.25	2	50	64
Lyophilized supernatant	7.1	19.5	23.5	39.05	3.22	4.8	14.15	39.1

^*a*^ The negative control consisted of the yeast culture without any inhibitor. The dashes indicate the lack of effect on the yeast strains in the absence of an inhibitor.

### *Candida* spp. growth recovery by adding mevalonate, squalene, or ergosterol

**Fig 5:**
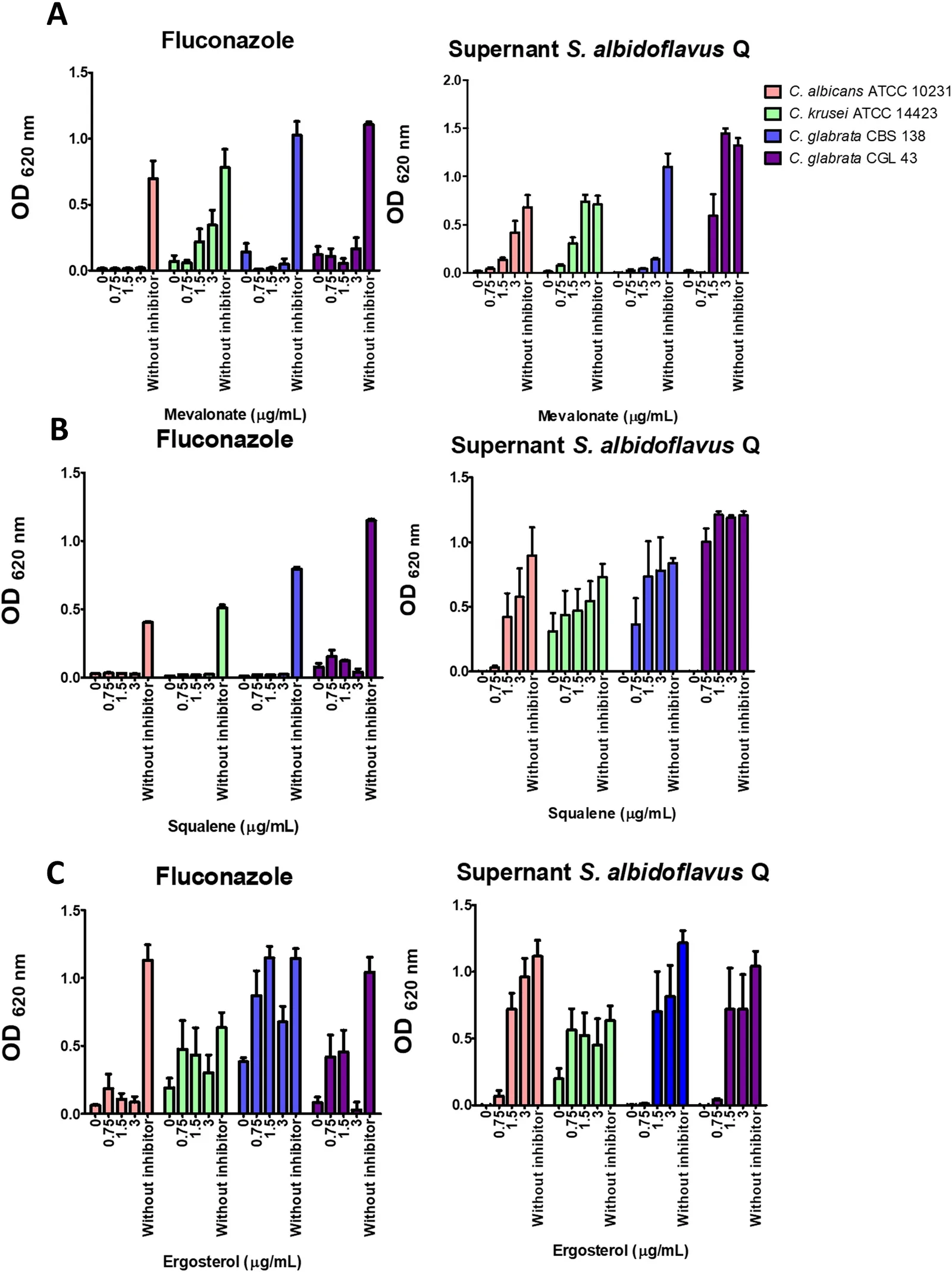
A *Candida* spp. growth rescue assay was carried out. The yeasts were first treated with fluconazole or the metabolites of *S. albidoflavus* at sublethal concentrations, followed by the addition of exogenous mevalonate, squalene, or ergosterol to the culture medium. Following both initial treatments, ergosterol caused *Candida* spp. to undergo growth recovery. Squalene and mevalonate promoted growth recovery of *Candida* spp. after treatment with *S. albidoflavus* metabolites but not after treatment with fluconazole. The yeasts without antifungal treatment served as the control. Yeast growth was quantified by optical density in a Thermo Scientific Multiskan FC microplate photometer at 620 nm (OD_620_) upon completion of incubation at 37°C for 24 h. The values are expressed as the average of three independent assays ± SD. The basal growth value was established with the control (the yeast without any inhibitor). Significant differences were analyzed by two-way ANOVA. ***P < 0.001.

A yeast growth recovery experiment explored the possibility that the secondary metabolites produced by *S. albidoflavus* Q affect ergosterol biosynthesis in *C. glabrata*. The antifungal metabolites were applied at the previously established sublethal concentrations (MIC_70-90_). The antifungal compounds inhibited the growth of the yeasts, but the addition of exogenous mevalonate, squalene, or ergosterol to the culture medium resulted in growth recovery for most yeast strains. In the case of the lyophilized supernatant as the antifungal, the growth recovery of the yeast increased with higher concentrations of exogenous mevalonate, squalene, or ergosterol. The recovery was not as evident with *C. glabrata* CBS 138 (Fig. 5). When the yeasts were treated with fluconazole, growth recovery was only observed with ergosterol, as expected (since the azoles act on the Erg11 enzyme, which is in the last stage of the ergosterol biosynthesis pathway). *C. albicans* ATTC 10231 did not show recovery with ergosterol.

### Effect of *S. albidoflavus* Q metabolites on ergosterol synthesis in *Candida* spp. species

**Fig 6:**
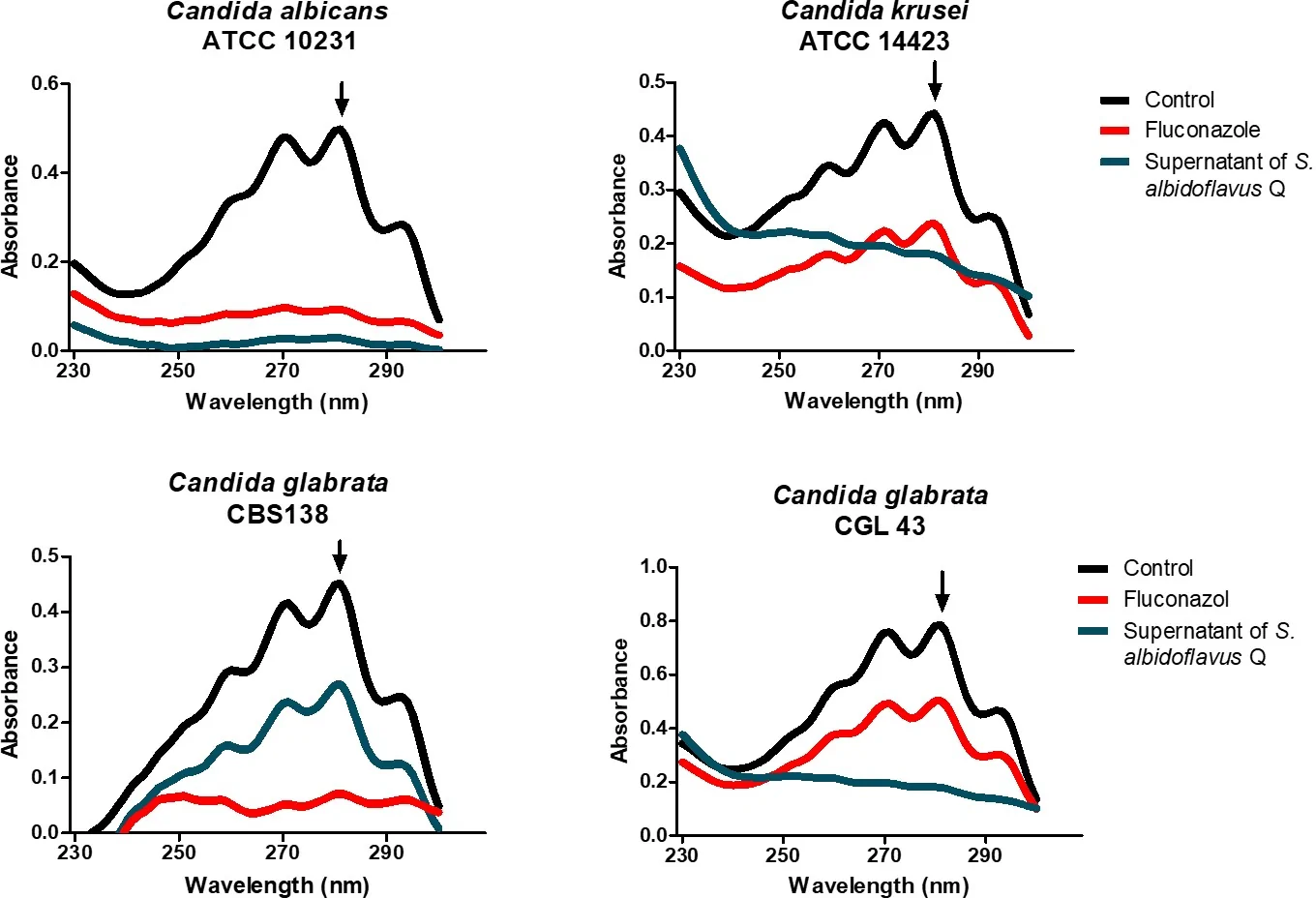
Effect of the lyophilized supernatant of *Streptomyces albidoflavus* Q on the concentration of ergosterol in *Candida* spp. species. The different species were grown in YPD liquid yeast medium and treated with sublethal concentrations of the inhibitors. The control was the growth of the yeasts in the absence of any inhibitor. The yeasts were incubated at 37°C for 18 h under constant shaking at 200  rpm. The concentration of the extracted sterols (in the n-heptane layer) was determined by spectrophotometrically scanning them (from 230 to 300  nm). The extraction of total sterols was performed on a 100 mg sample of yeast.

A possible association between the inhibition of growth and the concentration of ergosterol in *Candida* spp. species was examined with each of the two treatments (the lyophilized supernatant and fluconazole as the control). The absorption spectra of the control displayed the characteristic four peaks of sterols (Fig. 6). The lyophilized supernatant at sublethal concentrations caused reduced ergosterol levels in 100 mg samples of the yeasts.

The absorption peak at 281.5 nm was used to quantify the ergosterol concentration, allowing for the calculation of the percentage of inhibition of its synthesis. Compared to fluconazole, the metabolites of the supernatant of *S. albidoflavus* Q afforded a greater percentage of inhibition of ergosterol in the yeasts (Table 3). This result suggests that the target site of the metabolites is located at some stage of the ergosterol biosynthetic pathway.

**TABLE 3:** Percentage of ergosterol inhibition found in the distinct species of *Candida* spp. after treatment with fluconazole or the lyophilized supernatant of *S. albidoflavus* Q, or in the absence of treatment (the control)

	*C. albicans* ATCC 10231	*C. krusei* ATCC 14423	*C. glabrata* CBS 138	*C. glabrata* CGL 43
Control^*a*^	0	0	0	0
Fluconazole	60	15	41	29
Lyophilized supernatant	89	89	100	66

^*a*^ The control consisted of the yeast cultivated in the absence of an antifungal. Each *Candida* spp. was subjected to a sublethal concentration of fluconazole or the lyophilized supernatant of *S. albidoflavus* Q. The yeasts were incubated at 37°C for 18 h under constant shaking at 200  rpm.

### Effect of the *S. albidoflavus* Q supernatant on the cytoplasmic membrane structure of *C. glabrata*

**Fig 7:**
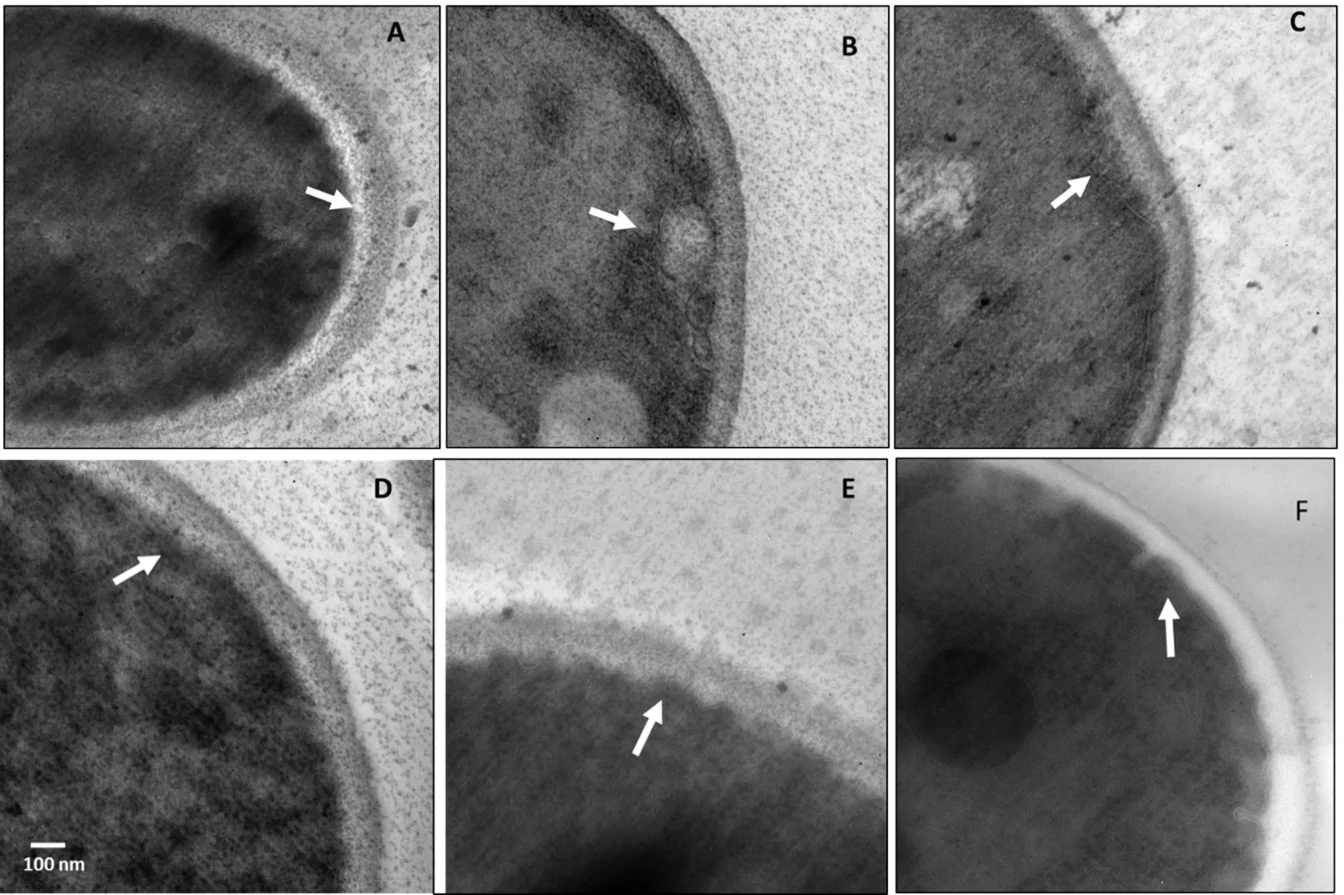
Image of *Candida glabrata* treated with the *S. albidoflavus* Q supernatant, taken with a transmission electron microscope. Micrographs: (**A–C**) of *C. glabrata* CBS 138 and (**D–F**) of *C. glabrata* CGL 43. (**A and D**) Without treatment (negative control); (**B and E**) treated with fluconazole (positive control); (**C and F**) treated with the lyophilized supernatant of *S. albidoflavus* Q. The arrows indicate the cytoplasmic membrane with (**C and F**) and without (**A and D**) treatment. The yeasts were each treated with sublethal concentrations of the antifungals at 37°C for 18 h in YPD liquid yeast medium.

Unlike the untreated control, yeasts treated with a sublethal concentration of fluconazole or the lyophilized supernatant of *S. albidoflavus* Q showed morphological changes. The yeasts were treated with inhibitors and examined by transmission electron microscopy (TEM). In the micrograph of the yeasts treated with the *S. albidoflavus* Q supernatant, the cellular membrane exhibited invaginations, probably caused by antifungal metabolites produced by the bacterium (Fig. 7). Fluconazole was used as the positive control.

### Toxicity of the supernatant tested in the *Galleria mellonella* model

The possible toxicity of the metabolites found in the supernatant of *S. albidoflavus* Q was evaluated by using the *Galleria mellonella* model. However, no toxicity was observed, as shown by the 50% DMSO toxicity control (Table S2).

### Metabolomics of *Streptomyces albidoflavus* Q

**Fig 8:**
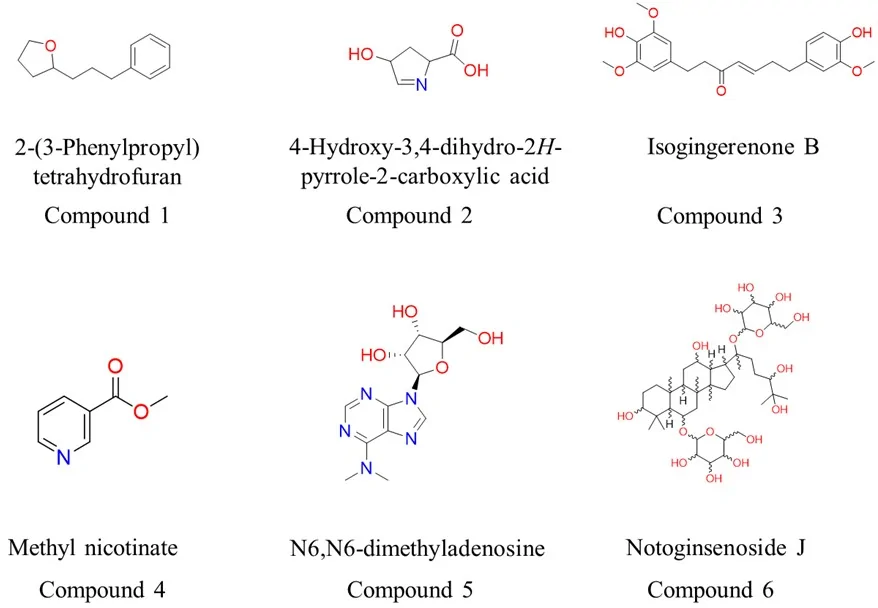
Structures of metabolites with potential inhibitory activity on *Cg*HMGR. First, 66 secondary metabolites were found in the lyophilized supernatant of *S. albidoflavus* Q through metabolomic analysis. Subsequently, they were examined by using the web resource PASS Online, deposited in server Way2Drug (http://way2drug.com/passonline/), which selected the most probable inhibitors.

Based on a metabolomic study of the lyophilized supernatant of *S. albidoflavus* Q, at least 66 metabolites were identified (Fig. S1). They were analyzed by the Way2drug server (http://way2drug.com/passonline/) to find potential inhibitors of *Cg*HMGR. The corresponding chemical structures are shown in Fig. 8.

### M-BGCs and metabolism in *S. albidoflavus* Q

Detailed analysis of the *S. albidoflavus* Q genome sequence using antiSMASH 4.0 and manual curation suggests the existence of 38 BGCs that encode diverse special metabolites (Table 4). Notoginsenoside J (compound 6; Fig. 8) has a triterpenoid structure similar to hopanoids. Hence, the metabolite bacterial core gene (M-BCG) present in the sequence of region 1.4 could possibly be related to the biosynthesis pathway of this compound. Other characteristics of the compound are discussed below.

**TABLE 4:** Known/predicted metabolites and the most similar BGCs in *S. albidoflavus* Q, determined with antiSMASH^*a*^

Region	Type	Most similar BGCs	Percent of similarity	MIBiG BGC ID
1.1	NPR-metallophore, NPRS	Griseobactin	100	BGC0000368
1.2	Terpene, NRPS	Valinomycin/Montanastatin	13	BGC0001043
1.3	T1PKS, NRPS	SGR PTMs/SGR PTM Compound b/SGR PTM Compound c/SGR PTM Compound d/	100	BGC0001043
1.4	Terpene	Hopene	76	BGC0000663
2.1	NRPS-independent siderophore	Desferriozamine B	100	BGC0000941
3.1	Terpene	Geosmin	100	BGC0001181
3.2	Terpene	Julichrome Q3-3/Julichrome Q3-5	25	BGC0002012
4.1	Terpene	Isprenieratene	75	BGC0001456
4.2	T3PKS	Valinomycin/ Montanastatin	34	BGC0001846
4.3	T1PKS	Candicidin	33	BGC0000034
6.1	NRPS, T2PKS	Fredericamycin A	93	BGC0000224
7.1	Lanthipeptide class III	AmfS	80	BGC0000496
9.1	NRPS	Cyclofaulkamycin	75	BGC0002358
10.1	T1PKS, NRPS, Lanthipeptide class II	Candicidin	95	BGC0000034
12.1	Ectoine	Ectoine	100	BGC0000853
20.1	LAP, NRPS	Surogamide A/Surogamide D	63	BGC0001792
28.1	NPRS	Surogamide A/Surogamide D	57	BGC0001792

^*a*^ BGC, biosynthetic gene cluster; MIBiG BGC ID, minimal information for the identification of a biosynthetic gene cluster; NRPS, non-ribosomal peptide synthetase; LAP, linear azol(in)e-containing peptides; T1PKS, type 1 polyketide synthase; T2PKS, type 2 polyketide synthase; T3PKS, type 3 polyketide synthase.

### Docking study of the secondary metabolites identified as potential inhibitors of *Cg*HMGR

**Fig 9:**
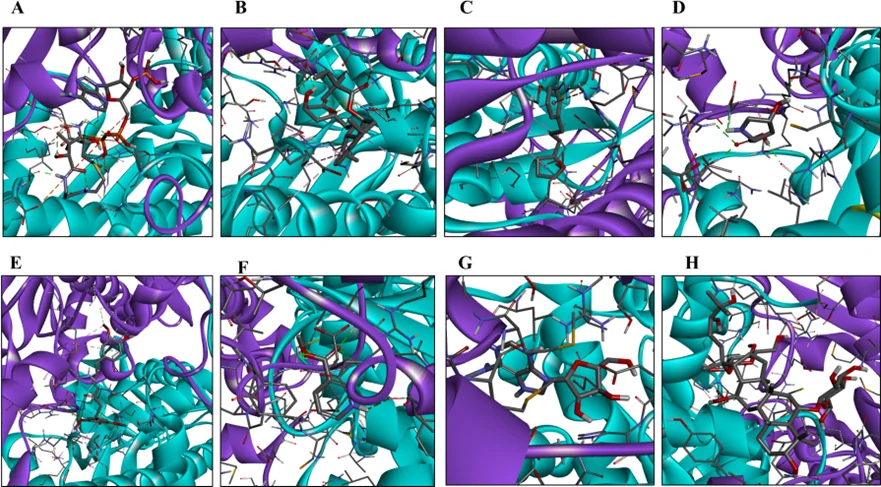
For each of the metabolites identified as potential inhibitors of HMGR, a schematic illustration portrays the binding mode of the ligand with the *Cg*HMGR enzyme. The predicted binding mode of HMG-CoA (**A**), simvastatin (**B**), compound 1 (**C**), compound 2 (**D**), compound 3 (**E**), compound 4 (**F**), compound 5 (**G**), and compound 6 (**H**). The α-helix and β-strand structures are depicted as ribbons, colored in cyan (subunit a) and purple (subunit b). The amino acids that interact with the ligand and the ligand itself are represented as sticks. The figure was created by Celia-Esthela Bautista-Crescencio with Discovery Studio 2021 client software.

A coupling analysis was performed to test the hypothesis of possible interactions between the antifungal metabolites of *S. albidoflavus* Q and *Cg*HMGR. The results show low binding energy values (reflecting a high affinity between the ligands and the protein), which would explain the inhibition of enzyme growth and, likewise, the decrease in ergosterol levels as well as alterations in the cytoplasmic membrane. First, the structure of the protein (*Ca*HMGR) was built in 3D and then verified. The theoretical model was validated with a Ramachandran plot for *C. albicans* HMGR (*Ca*HMGR) and *Cg*HMGR. Of the total amino acids, those in the favorable region constituted 90.1% and 93.1%, respectively, which indicates good reliability of the structure (Fig. S2). Subsequently, the 3D structures were used for docking studies to explore the recognition of *Cg*HMGR by the secondary metabolites. The 3-hydroxy-3-methyl-glutaryl-CoA substrate (HMG-CoA) of the HMGR enzyme was docked with a known inhibitor, simvastatin, as the binding control. The theoretical interaction of each secondary metabolite with *Cg*HMGR is expressed as the binding energy (the values are listed in Table 5). The schematic binding mode of the ligands with the enzyme is illustrated in Fig. 9. Isogingerenone B was the metabolite with the highest binding energy. Its binding to the enzyme was based on hydrogen bonding, Van der Waals forces, and π-sigma, π-anion, and alkyl interactions. Notoginsenoside J showed the second highest binding energy, followed by N6,N6-dimethyladenosine.

**TABLE 5:** Docking results of the binding mode between metabolites produced by *S. albidoflavus* Q at the catalytic site of the *Cg*HMGR enzyme^*a*^

Molecules	Bindig energy (kcal/mol)	Interacting residues	Interactions
3-Hydroxy-3-methylglutaryl CoA (HMG-CoA)	−9.1	Asn A: 810, Ile A: 956, Gly A: 919, Gln A: 920, His B: 906, Asn B: 909, Leu B: 1013, Asn A: 810	Conventional hydrogen bond
		Cys A: 678, Ala A: 677, Thr B: 710, Gly A: 808, Met A: 811, Gly A: 957, Gly A: 959, Gly A: 954, Met A: 807, Asp A: 921, Val A: 953, Thr A: 960, Ser A: 813, Met A:809, Ala B: 1007, Ser B: 717, Lys A: 845, Asp A:844, Leu B: 1004, Leu B: 1008, Leu B: 714, His B: 1012, Val B: 1014.	Van der Waals
		Gly A: 958, Cys B: 713.	Carbon-hydrogen bond
		Glu B: 711, Gln B: 1015, Lys B: 889, Arg A: 742.	Attractive charge
		Ala A: 806.	Pi-Alkyl
Simvastatin	−8.2	Asn A: 810, Asn B:909, Asp A: 844, Arg A: 742.	Conventional hydrogen bond
		Cys B: 713, Leu B: 1013, Ala B: 1007, Leu B 1004	Alkyl
		Val B: 1014, Gly B: 712, Gln B:1015, Leu B: 714, Glu B: 711, Ser B:717, His B: 906, Lys A: 846, Lys A: 845, Ala B:905, Ser A: 838, Met A: 809, Ser A: 209.	Van der Waals
2-(3-Phenylpropyl)-tetrahydrofuran (Compound 1)	−6.2	Lys B: 889, Lys A: 846, Ala B:905, Lys A: 845, Asn B: 909, Glu B: 711, Leu B: 714, Ser B: 717, Cys B: 713, Gly B: 712, Leu B: 1013, Ser A: 838	Van der Waals
		Arg A: 742, Asp A: 844	Pi Anion/Cation
		Leu B: 1004	Pi-sigma
		His B: 906, Leu B: 1004	Alkyl
4-Hydroxy-3,4-dihydro-2H-pyrrole-2-carboxylic acid (Compound 2)	−5.4	Lys B: 889, Lys A: 846, Leu B: 1004, His B: 906, Lys A: 845, His B: 906, Lys A: 845, Asn B:909, Leu B: 1013.	Van der Waals
		Ser A: 838, Ala B: 905, Arg A: 742, Asp A: 844, Glu B: 711	Conventional hydrogen bond
Isogingerenone B (Compound 3)	−8.3	Asn B: 909, His B: 906, Arg A: 742	Conventional hydrogen bond
		Lys A: 845, Ser A: 838, Asp A: 844, Asp A: 921, Thr B: 710, Thr A: 960, Gly A: 919, Gly A: 957, Gly A: 958.	Van der Waals
		Leu B: 714, Leu B: 1004, Met A: 809, Met A: 807.	Alkyl
		Leu B: 1013	Pi-Sigma
		Glu B: 711	Pi-Anion
Methyl nicotinate (Compound 4)	−5.8	Tyr A: 630, **Val A: 682, Asn A: 681**	Conventional hydrogen bond
		Glu A: 630, Arg A: 646, Tyr A: 671, Phe A: 675, **Glu A: 680**, Gly A: 676, Cys A: 679, Val A: 674.	Van der Waals
N6, N6-dimethyladenosine (Compound 5)	−7.6	Asp A: 844, Lys B: 889, Ala B: 905.	Conventional hydrogen bond.
		Glu B: 711, Leu B: 1013, Gly B: 712.	Carbon-hydrogen bond
		His B: 906, Ser A: 838, Leu B: 1008, Asn A: 840, Arg A: 742, Leu B: 1004, Arg A: 742, Met A: 809*, Ser A: 813. Asn A: 810*, Cys B: 713, Asn B: 909, Lys A: 845, His B: 906.	Van der Waals
Notoginsenoside J (Compound 6)	−7.8	His B: 1012, Ser B: 717, Cys B: 713	Conventional hydrogen bond
		His B: 876	Pi-donor hydrogen bond
		Arg B: 720, Ser B: 1003, Leu B: 1004, Ala B: 1007, Leu B: 1013, Gln B: 1015, Met B: 719, Ala B: 716, Tyr A: 630, Asn A: 681, Glu A: 633, Phe A: 675, Lys A: 679, Gly A: 676, Ala A: 677. Gly B: 712, Lys B: 876,	Van der Waals

^*a*^ The binding energy is expressed as kCal/mol (ΔG). HMG-CoA, the natural substrate of the enzyme HMGR, and simvastatin were used as binding controls. Modeling analysis of the CgHMGR protein suggests a dimer conformation. The marked aa correspond to the A or B chain of the dimer. The aa belonging to the **ENVIG** dimerization motif are indicated in **bold,** the aa of the EGCLVAS substrate binding motif are underlined, and the aa of the DAMGMN* cofactor binding motif are indicated with an asterisk.

## DISCUSSION

The emergence of multidrug-resistant strains of fungi and their ability to cause nosocomial outbreaks of infection in immunocompromised patients has become an increasingly severe problem. Another global challenge that has emerged in the last couple of years is the appearance of polymicrobial infections (viruses, bacteria-fungi) associated with SARS-Cov-2/COVID and HIV/AIDS (2, 26). The emergence of drug-resistant strains of *Candida* is particularly problematic due to the limited existing treatment options, consisting of only a few antifungal drug families (27). The discovery of new antifungals with alternative therapeutic targets should contribute to the mitigation of these problems.

Regarding antifungal metabolites from actinobacteria, some are already clinical drugs (e.g., amphotericin B, nystatin, and natamycin) (Table 1). The development of new antifungal compounds derived from microorganisms faces the challenge of defining the chemical structure of such molecules as well as their therapeutic target and binding mode. Moreover, the identification of species of *Streptomyces* strains capable of generating antifungal metabolites can be complicated by the necessity of examining more than four molecular markers (28). The latter problem has been resolved with phylogenomic analysis, which is based on whole-genome sequencing (WGS) with a 92-gene core. By means of this technique, *S. albidoflavus* was established as the species of *Streptomyces* Q responsible for producing the present antifungal compounds.

Since one of the objectives of this study was to search for inhibitors of the HMGR enzyme, a growth recovery assay was carried out to try to infer which block of enzymes of the ergosterol synthesis pathway is involved in the inhibition caused by the S. *albidoflavus* Q metabolites (Fig. 1). The growth recovery of *C. glabrata* with mevalonate, squalene, and ergosterol suggests that the S. *albidoflavus* Q metabolites affect the first module of the ergosterol biosynthetic pathway (Fig. 1) (21, 23, 24, 29). *C. albicans* ATCC 10231 did not show growth recovery with ergosterol, perhaps because of the distinct sterol uptake responses between yeasts like *Schizosaccharomyces pombe, C. glabrata*, and *C. albicans* (30). On the contrary, the difference between the *C. glabrata* CBS 138 and CGL 43 strains in regard to growth recovery with mevalonate might be associated with the distinct genetic background of the two strains, the first of which is susceptible and the second is resistant to fluconazole (23). The regulation of the ergosterol biosynthesis pathway could vary from one *Candida* species to another (24, 30).

Considering the growth recovery with mevalonate, the decrease in ergosterol levels (Table 2), and the alteration of the cytoplasmic membrane of *C. glabrata*, the secondary metabolites produced by *S. albidoflavus* Q likely act by inhibiting the ergosterol biosynthesis pathway and in particular the *Cg*HMGR enzyme (31). The latter enzyme is a key and limiting step in ergosterol synthesis (32) (Fig. 1).

Therefore, a selection process was carried out to identify the probable candidates for the inhibition of ergosterol among the 66 compounds identified by LC-HRMS metabolomic analysis. Six compounds with the greatest probability of interacting with the *Cg*HMGR enzyme were selected as possible inhibitors by using the Way2Drug server (http://way2drug.com/passonline/) (Fig. 8). These secondary metabolites were tested *in silico* for their capacity to bind to the *Cg*HMGR enzyme. According to the docking study (Fig. S1), the secondary metabolites isogingerenone B (compound 3) and notoginsenoside J (compound 6) interact with *Cg*HMGR with high binding energy.

Isogingerenone B produced by *Zingiber officinale* was proposed in a previous report as a potential antifungal compound (33). However, the molecular target was not explored and its antifungal activity was not evaluated in *Candida* strains resistant to azoles.

In another study, molecular modeling analysis suggested that isogingerenone may bind to the fatty acid synthase enzyme, a marker protein in acute lymphoblastic leukemia (34). It has also been documented that this compound acts preferentially on senescent cells and thus has senolytic activity (35). Finally, isogingerenone B has the structure of a diarylheptanoid (https://pubchem.ncbi.nlm.nih.gov/compound/Gingerenone-B), closely related to the structures of curcumins and flavonoids. The applications of the latter compounds of plant origin have been widely documented in medicine and food processing (36). Overall, this evidence warrants further research on the pharmaceutical applications of isogingerenone B derived from *S. albidoflavus* Q.

The *S. albidoflavus* Q strain was herein isolated from rhizospheric maize soil in Mexico (37). Although the role of *S. albidoflavus* Q metabolites in the ecology of the bacterium is outside of the scope of the current contribution, there is a relevant precedent: the relation of antifungal compounds of *Streptomyces griseus* with the control of the phytopathogenic fungus *Fusarium. S. griseus* S4–7 was initially isolated from the strawberry rhizosphere as a microbial agent responsible for suppressing *Fusarium* wilt in soil (38). Among the genes of *S. griseus* proposed to participate in the antifungal activity against *Fusarium* are those linked to the synthesis of diarylheptanoid and gingerol, compounds closely related to the chemical structure of isogingerenone B of *S. albidoflavus* Q. Thus, it would be interesting to investigate the effect of antifungal *S. albidoflavus* Q metabolites on phytopathogenic fungi of maize. In this sense, there are reports on polyphenolic metabolites of *Streptomyces clavuligerus* (associated initially with plants) and their possible biosynthetic routes, identified through genetic engineering techniques and special conditions for expressing some cryptic genes (39).

Notoginsenoside J (compound 6), the other *S. albidoflavus* Q metabolite identified as a potential antifungal agent, has been previously evaluated for antioxidant (40), anti-tumor, anti-fatigue, anti-inflammatory (41), and anti-atherosclerosis activities (42). However, the molecular target of notoginsenoside is unknown (43). According to the present analysis, the *Cg*HMGR enzyme is a plausible target. Notoginsenoside J is a glycosylated triterpenoid with a chemical structure similar to the *Streptomyces* hopanoids (44, 45). Polycyclic triterpenes, members of the terpene family, are synthesized by the cyclization of squalene, and the biosynthesis pathway is highly conserved in bacteria and certain eukaryotes (46). Hence, it would not be surprising if the production of hopanoids and notoginsenosides share some enzymes of the same biosynthesis pathway. The biosynthesis pathway of triterpenoids in actinobacteria has been defined and is related to the mevalonate pathway (47). Some genes involved in this pathway have been identified in *S. avermitilis* (https://www.genome.jp/kegg-bin/show_pathway?sma00909). The enzymes that putatively participate in the glycosylation of triterpenoids in *S. clavuligerus* have also been described (39). The BGCs contained in *S. albidoflavus* Q were herein analyzed to explore their biosynthetic capacity by matching them with the metabolites detected in this study (Table 4). Subsequently, some putative terpenoid pathways in the *S. albidoflavus* Q genome were established with the antiSMASH tool. A putative squalene cyclase was identified and related to the biosynthesis of hopenes in the *S. albidoflavus* Q genome (https://mibig.secondarymetabolites.org/repository/BGC0000663/index.html#r1c1).

On the contrary, it has been observed in yeasts (as in mammals) that sterol synthesis is highly regulated at the transcription, translation, and post-translation levels, as is the inhibition of sterols by the intermediate or final products of the enzymatic activity of the biosynthesis pathway (Fig. 1) (24, 31). Therefore, it would be interesting to examine the role of triterpenoids from *S. albidoflavus* Q in regulating the enzymes involved in the synthesis of ergosterol in yeasts and in the regulation of triterpenoid synthesis in actinobacteria.

Isogingerenone and notoginsenosides have been associated mainly with plants (33, 43), but compounds such as coumarins have also been identified in the extracts of *Streptomyces* spp. by metabolomic analyses (48). Naringenin, another antifungal compound that was initially known for its plant origin, is generated by *Streptomyces clavuligerus* (39, 49).

In the current *in silico* study, N6,N6-dimethyladenosine (compound 5) showed the third highest binding free energy when docked with *Cg*HMGR. It has been used as an inhibitor of AKT signaling in various lung cancer cell lines (50). In Actinomycete *Mycobacterium bovis*, this molecule may be responsible for the post-transcriptional modification of tRNAs (51). Further research is necessary on N6,N6-dimethyladenosine considering the scant number of reports on this molecule in the literature. The possible interaction of this metabolite with the amino acids of the NADPH cofactor binding motif of the *Cg*HMGR protein is discussed below.

The docking analysis of six *S*. *albidoflavus* Q metabolites gives important clues as to their binding mode (Table 4). The three most conserved motifs for the protein of fungal HMGR enzymes are ENVIG dimerization (680–684 residues), EGCLVAS substrate binding (711–717 residues), and cofactor DAMGMN binding (807–812 residues). The role of these amino acids in the activity of *Cg*HMGR has also been assessed through the evaluation of point mutants in each of the enzyme motifs (22).

Methyl-nicotinate (compound 4) might affect the dimerization of *Cg*HMGR given its interaction with the amino acid residues of the ENVIG dimerization motif of the enzyme. 2-(3-Phenylpropyl)-tetrahydrofuran, 4-hydroxy-3,4-dihydro-2*H*-pyrrole-2-carboxylic acid, isogingerenone B, and notoginsenoside J (compounds 1, 2, 3, and 6, respectively) may interact with the amino acid residues of the EGCLVAS substrate-binding domain, as well as with the control molecules HMG-CoA (an HMGR substrate) and simvastatin (an HMGR inhibitor).

The docking analysis suggests that the secondary metabolite N6,N6-dimethyladenosine (compound 5) interacts with some amino acids of the DAMGMN cofactor-binding motif of the enzyme (22). Hence, this compound could possibly compete with NADPH, the natural cofactor of the enzyme. Regarding compound 2, synthetic substituted pyrroles are known to act as antifungals by inhibiting *Cg*HMGR activity and ergosterol synthesis (23).

There are few reports on the strains of *S. albidoflavus* that produce antifungal metabolites. The *S. albidoflavus* C247 strain was isolated from soil samples in Korea, but its identification was based only on the sequence of the 16S rDNA molecular marker, and its antifungal activity was determined by inhibition of the formation of *Rhizoctonia solani* mycelium (52). A non-polyene antifungal antibiotic from *S. albidoflavus* PU 23 was also described (53).

Since ergosterol is an essential constituent of the fungal plasma membrane (54), the inhibition of one or more key enzymes of the ergosterol biosynthesis pathway has been considered as an important element for antifungal drugs (24). Squalene epoxidase (Erg 1) targets allylamines, a noncompetitive inhibitor (55). Azoles, the most common antifungal for treating invasive fungal infections, target the lanosterol 14 alpha-demethylase enzyme (Erg11) (56). Polyenes interact with the ergosterol surface and form pores in the plasmatic membrane (57). Micrographs of the cytoplasmic membrane of *C. glabrata* treated with sublethal concentrations of the supernatant of *S. albidoflavus* Q evidence alterations in the cytoplasmic membrane of the yeasts, similar to those caused by fluconazole, an ergosterol inhibitor (56, 58).

According to the results of the current contribution, *Cg*HMGR is probably the molecular target of antifungal *S. albidoflavus* Q metabolites, which inhibited the growth of *C. glabrata*, diminished its ergosterol synthesis, and altered its cytoplasmic membrane. These metabolites were identified and used in molecular modeling, which showed their interaction with the *Cg*HMGR enzyme.

In the future, it will be necessary to carry out metabolomics studies on yeasts treated with *S. albidoflavus* purified metabolites capable of inhibiting the HMGR enzyme or other enzymes of the ergosterol biosynthesis pathway. The synthesis of sterols in mammals and yeasts is a highly regulated process through all stages of the flow of genetic information and at the biochemical level (32). Metabolomic studies on the effect of fluconazole treatment of *C. albicans* have shown an increase in the central carbon and a decrease in the synthesis of intermediate amino acids, suggesting a rerouting of metabolic pathways. The function of these metabolomic changes is still not clear. As aforementioned, they may represent previously unrecognized mechanisms of the metabolic alteration of *C. albicans* induced by fluconazole (59).

The metabolites present in the supernatant of *S. albidoflavus* Q did not show toxicity, according to the *Galleria mellonella* model currently used. However, it will be necessary to evaluate possible toxicity when the compounds with antifungal activity have been purified and their activity and potency verified.

### Conclusion

The species of actinobacteria isolated from rhizospheric maize soil was identified as *S. albidoflavus*. The genome of *S. albidoflavus* Q exhibited characteristics typical of an actinobacterial genome: a high percentage of G+C and linear topology. The sequence herein generated and deposited in the Genbank (PRJNA886754) will allow for further analysis of the WGS. The biosynthetic pathways of the metabolites of interest can then be established through metabolic engineering, expression analysis, and the development of mutants to verify the genes involved in the synthesis of the antifungal compounds.

It was presently demonstrated that *S. albidoflavus* Q produces secondary metabolites with antifungal activity, evidenced by reduced yeast viability, decreased ergosterol level, and alterations in the yeast cytoplasmic membrane ultrastructure. Such effects are carried out by targeting the ergosterol pathway and probably the HMGR enzyme. This conclusion is based on the decrease in the level of ergosterol and the posterior growth recovery of *Candida* spp. with mevalonate, squalene, and ergosterol. The secondary metabolites isogingerenone B (compound 3) and notoginsenoside J (compound 6) interact *in silico* with the substrate-binding EGCLVAS motif of the *Cg*HMGR protein with high binding energy, supporting the *in vitro* experimental results.

After identifying potential antifungal compounds, their rational design involves chemical modifications to achieve more potent, more target-specific, and less toxic molecules, followed by their *in vitro* testing on *Candida* species. Subsequently, the derivatives can be examined for their safety, toxicity, pharmacokinetics, and pharmacodynamics, and finally tested in clinical trials.

## MATERIALS AND METHODS

### Strain and culture media

The *Streptomyces* Q strain belongs to a collection of around 300 isolates of actinobacteria from different soils and Mexican plants, which is stored in the Molecular Biology of Bacteria and Yeast Lab of the Escuela Nacional de Ciencias Biológicas of the Instituto Politécnico Nacional in Mexico. This strain was selected for its ability to inhibit the growth of *C. glabrata*.

*S. albidoflavus* Q was grown in GAE medium (2% glucose, 1% asparagine, 0.5% yeast extract, 0.5% K_2_HPO_4_, 0.5% MgSO_4_, and 0.01% FeSO_4_) for 15 days at 28°C under constant agitation at 250 rpm. Subsequently, the biomass was separated by centrifugation at refrigeration temperature, and the supernatant was lyophilized. The resulting supernatant was solubilized in sterilized ultrapure MilliQTM water and then concentrated at 100 mg/mL (wt/vol). The morphology and purity of the colony of *Streptomyces* Q were verified on solid GAE medium (2% bacteriological agar).

*C. glabrata* CBS 138 and CGL 43 (phenotypes susceptible and resistant to fluconazole, respectively) were employed to examine the antifungal effect and ergosterol inhibition promoted by the *S. albidoflavus* Q metabolites. The *C. glabrata* CBS138 used in this study was kindly provided by Bernard Dujon from the Instituto Pasteur-Paris. *C. glabrata* CGL 43 belongs to the collection of our laboratory and was isolated from a patient with HIV from the Civil Hospital of Guadalajara and donated by Dr. Fernando Velarde Rivera. On the contrary, *C. albicans* ATCC 10231 and *C. krusei* ATCC 14423 served as the control of susceptibility and resistance to fluconazole, respectively. The yeasts were grown in extract-peptone-dextrose YPD liquid yeast medium (1% yeast extract, 2% dextrose, and 2% casein peptone) at 37°C under constant agitation at 250 rpm. The strains were stored at –70°C in 50% (vol/vol) anhydrous glycerol (Sigma-Aldrich) to await the testing of the metabolites as fungal inhibitors.

### Whole-genome sequencing (WGS) of *Streptomyces albidoflavus* sp Q and phylogenetic analysis

After the *S. albidoflavus* Q strain was grown, the DNA was extracted with the Zymo Research Soil Microbe DNA Miniprep kit. The whole-genome sequencing was performed by using Illumina Hiseq4000 (Novogene, Sacramento, CA, USA). The reads underwent trimming with Trimmomatic (60) before conducting a search for the sequences of the PhiX phage in the genome of the *Streptomyces* Q (61). The genome was assembled on SPAdes software (62), and the assembly quality was evaluated with the QUAST program (63).

The phylogenomic tree of *Streptomyces* spp. was created by aligning 92 genes (core genome) on UBCG software (64). The genome sequences of the following strains were taken as an outgroup from NCBI: *S. albidoflavus* strain DSM 40455 (NZ_PKLO01000004.1), *S. albidoflavus* strain J1074 (CP004370.1), *S. albidoflavus* strain J1074/*R2* (GCA_013693715.1), *S. albidoflavus* strain NBRC 100770 (PKLL01000003.1), *S. albidoflavus* strain NBRC 12790 (PKLN01000043.1), *S. albidoflavus* strain R-53649 (FWFA01000334.1), *S. albidoflavus* strain SM254 (NZ_CP014485.1), *S. albovinaceus* strain NRRL B-2566 (NZ_MUAX01000001.1), *S. albus* strain NBRC 13014 (NZ_BBQG01000095.1), *S. antibioticus* strain DSM 41481 (NZ_CM007717.1), *S. aquilus* strain GGCR-6 (NZ_CP034463.1), *S. argenteolus* strain 3259 Ga0365441_101 (NZ_VIWQ01000001.1), *S. aureus* strain NRRL B-2808 (NZ_LIPQ01000001.1), *S. bobili* strain NRRL B-1338 (NZ_MUBA01000001.1), *S. fungicidicus* strain TXX3120 (NZ_CP023407.1), *S. finlayi* strain NBSH44 (NZ_CP045702.1), *S. globisporus* C-1027 (NZ_CP013738.1), *S. hygroscopicus* strain XM201 (NZ_CP018627.1), *S. hygroscopicus* subsp. *hygroscopicus* strain OsiSh-2 (NZ_MDFG01000001.1), *S. kanamyceticus* strain ATCC 12853 (NZ_CP023699.1), *S. lividans* TK24 (NZ_CP009124.1), *S. mediolani* strain NRRL WC-3934 (NZ_JOJK01000001.1), *S. microflavus* strain CG 893 (NZ_OAOR01000042.1), *S. mutomycini* strain NRRL B-65393 (NZ_MAPV01000001.1), *S. niveus* strain SCSIO 3406 (NZ_CP018047.1), *S. noursei* ATCC 11455 (NZ_CP011533.1), *S. rimosus* strain ATCC 10970 (NZ_CP023688.1), *S. sporangiiformans* strain NEAU-SSA 1 C1723 (NZ_VCHX02000184.1), *S. tsukubaensis* strain NRRL 18488 (NZ_CP029197.1), *S. venezuelae* ATCC 10712 (NZ_CP029197.1), and *Mycobacterium tuberculosis* H37Rv (NC_000962.3).

### Prediction of the biosynthetic gene clusters that encode the metabolites

Specialized metabolite biosynthetic gene clusters M-BGC were predicted with antiSMASH 4.0. c (65).

### Antifungal activity of the lyophilized supernatant of *S. albidoflavus* Q on *Candida* spp.

The modified microplate dilution method described by the Clinical and Laboratory Standards Institute (CLSI) was used to determine the minimum inhibitory concentration (MIC) of the lyophilized supernatant of *S. albidoflavus* Q (23, 66). Briefly, stock solutions were prepared with the lyophilized supernatant in RPMI 1640 medium (Sigma-Aldrich). The yeasts were then grown in YPD liquid yeast medium at 37°C for 24 h. The pre-inoculum OD_620_ = 0.6 was established before performing serial 1:1,000 dilutions to obtain the inoculum. Solutions of the supernatant or fluconazole (the inhibition control) were placed in microplates and incubated at 37°C for 24 h. The absorbance was read in a Thermo Scientific Multiskan FC microplate spectrophotometer at 620 nm (23).

### Antifungal activity of *S. albidoflavus* supernatant by disk diffusion

Yeast antifungal susceptibility was tested by disk diffusion in SDA medium. Fluconazole and water were used as the inhibition control and non-inhibition control, respectively. Sub-lethal concentrations were tested in the disks. The yeast was incubated at 37°C for 24 h, according to the CLSI protocol (modified) (https://clsi.org/standards/products/microbiology/documents/m44/).

### *Candida* spp. growth recovery with mevalonate, squalene, and ergosterol

A growth recovery experiment was carried out to verify that the lyophilized supernatant affects yeast viability by inhibiting ergosterol biosynthesis. Another objective of this experiment was to try to infer the enzyme or block of enzymes inhibited by the metabolites present in the supernatant of *Streptomyces* Q. Thus, the yeasts were treated with sublethal concentrations (MIC_70-90_, determined by the CLSI M27-A3 protocol) of an inhibitor, followed by the addition of mevalonate, squalene, or ergosterol (Fig. 1). Briefly, to each well of the microplates was added the concentration corresponding to the MIC_70-90_ of each inhibitor (the lyophilized supernatant of *S. albidoflavus Q* or fluconazole), which was elaborated with 80 µL of a yeast suspension adjusted to 1 to 5 × 10^6^ UFC/mL and diluted 1:1,000 with RPMI 1640 medium (Sigma-Aldrich). Next, a stock solution was prepared for each of the growth recovery compounds (mevalonate, squalene, and ergosterol; Sigma-Aldrich) by dissolving 120 µg/mL in Tween 80/ethanol (1:1) (Sigma-Aldrich). Subsequently, 20 µL of this solution was added to each well, resulting in a final concentration of 6, 3, or 1.5 µg/mL of mevalonate, squalene, or ergosterol, respectively. The controls consisted of yeast cultures treated with the vehicle only (in the absence of any antifungal compound, the growth control) and those treated with an antifungal but without sterol (the growth recovery control) (23).

The statistical analyses were performed, and graphs were constructed with GraphPad Prism 5.0. After calculating the mean of three replicates ± SD, differences between groups were examined with two-way analysis of variance (ANOVA), using the Bonferroni correction and a 95% confidence interval. Statistical significance was considered at *P* < 0.001 (22, 23, 29).

### Quantification of ergosterol in *Candida* spp.

Total sterols were extracted with a slightly modified version of the methodology reported by the authors of the references (67, 68, 68). Briefly, *Candida* spp. yeasts were grown in YPD yeast medium by incubation at 28°C for 24 h under constant agitation at 200 rpm. The cell culture was prepared by adjusting it to an optical density of 0.3 (AS_620_) in a different flask containing 5 mL of YPD yeast medium and then adding ultrapure MilliQTM water to control the lyophilized supernatant (IC_70-90_). Simvastatin and fluconazole served as the inhibition controls. For each treatment, both yeasts were incubated at 37°C for 18 h under constant shaking at 200 rpm. The cells were harvested by centrifugation and washed with sterile distilled water. Subsequently, each tube was adjusted to 100 mg of total yeast cells (wet weight) before adding 3 mL of an alcoholic potassium hydroxide solution (25 g of KOH and 35 mL distilled water, brought to 100 mL with absolute ethanol) in a vortex for 1 min to extract the sterols. The cell suspensions were incubated at 85°C for 1 h. The sterols were extracted with 1 mL of sterile distilled water and 2 mL of n-heptane by vigorously mixing the solution in a vortex for 3 min. The n-heptane layer was spectrophotometrically scanned between 230 and 300  nm (BioSpectrometer, Eppendorf). The presence of ergosterol (the As281.5 peak) and 24 dihydroxy-ergosterol (the As230 peak) [24 (28) DHE], a late intermediate, can be appreciated by the characteristic four-peaked spectrum indicating sterol absorption. This technique is also able to reveal a decrease in the level of ergosterol. The absence of detectable levels is evidenced by a flattening of the curve (23, 67 – 69).

### Transmission electron Microscopy of *Candida glabrata*

The antifungals at MIC_70-90_ were added to the culture media of *C. glabrata* CBS 138 and CGL43. Untreated cells served as the control for healthy membrane maintenance, and cells treated with fluconazole served as the control of cellular damage. The cells were centrifuged and washed twice with PBS Sörensen, then fixed by incubation with 2.5% glutaraldehyde for 1 h, post-fixed with 1% OsO_4_ in PBS at 4°C for 30 min, thoroughly washed with PBS, gradually dehydrated in an increasing ethanol series, and embedded in Spurr resin. The resin was polymerized at 60°C for 24 h and sectioned on an ultramicrotome Leica Ultracut R apparatus (Leica Microsystems, Heidelberg, Germany). The sections were recovered on copper grids, stained with uranyl acetate and lead citrate, and examined under a Jeol 2000EX transmission electron microscope (Jeol, Tokyo, Japan) at 80 kV (70).

### Metabolomic analysis with UHPLC-MS/MS data acquisition

For the UHPLC/Q-TOF-MS study, about 10 mg of the lyophilized supernatant of *S. albidoflavus* strain Q was placed in an Agilent 1290 Infinity II system coupled with a 6545A Q-TOF with a dual AJS ESI source (Agilent Technologies, Santa Clara, CA, USA), which was used in the positive mode in a standard mass range (m/z 3,200). The elution was carried out at 25 ± 0.5°C by using water (solvent A) and methanol (solvent B) with the following gradient: 100%A from 0 to 7 min, 40%A from 7 to 10 min, and 100%A with a 3 min re-equilibration time. The flow rate of the mobile phase was 0.6 mL/min with an injection volume of 20 µL for each sample. The organic phase was separated in an Agilent Zorbax AQUA, 4.6 × 150 mm^2^ column for 5 µM particles. During all LC-MS/MS analyses, samples were kept at 4°C. The raw data obtained by UHPLC/Q-TOF-MS/MS were converted into mzData by means of Agilent MassHunter Workstation Software Qualitative Analysis, version B.07.00, build 7.7.7024.29 SP2 (Agilent Technologies, Santa Clara, CA, USA). The parameters selected were those available for a UHPLC/UHD Q-TOF MS/MS, which included an m/z tolerated deviation of 15 ppm, a peak width from 5 to 20 s, a signal/noise threshold of 6, and an mzdiff of 0.01, mzwid of 0.015, bw of 5, and minfrac and minsamp of 0.5 and 1, respectively (71).

The secondary metabolites identified were analyzed for possible biological activity with the web resource PASS Online deposited in Way2Drug server (http://way2drug.com/passonline/) (72). Way2drug is a resource made up of various web services that are useful for the prediction of bioactivity of low molecular weight organic compounds. By simply drawing the chemical structure or entering the InChI code or Smiles code, the program performs the calculations of two parameters, Pa and Pi, that represent the probability of activity and inactivity of a compound, respectively. It is based on the local correspondence premise, according to which the biological activity of a drug-like organic compound may have effects similar to other compounds with a like structure. Using this concept, they developed a consistent system of neighborhood-focused atom descriptors including MNA Way2drug has been implemented in various SAR/QSAR/QSPR modeling approaches (72).

### Docking of the secondary metabolites identified as potential inhibitors of *Cg*HMGR

Homology modeling was performed by the Swiss-Model server (73), utilizing the sequence of HMGR of *C. glabrata* (Cg-HMGR, NCBI accession number XP_449268.1) and the crystallographic structure of human HMGR as a template (PDB entry 1DQ9, resolution 2.8 Å). The quality of the model was evaluated by determining the stereochemical restrictions with the Ramachandran plot constructed on PROCHECK (74). The protein preparations were obtained by following a standard protocol for the removal of co-crystallized ligands and water molecules. Polar hydrogens were added for all receptor atoms and computed to assess hydrogen-bonding interactions. All the other parameters were kept at their default settings (75). The structure was energetically minimized and equilibrated by means of molecular dynamic simulations on Autodock tools. The three-dimensional structure of the ligands was drawn in ChemSketch (www.acdlabs.com) and then subjected to energy optimization and minimization with GaussView 5.0 (76). The catalytic pocket was calculated with the server CASTp (http://sts.bioe.uic.edu/castp/index.html?3trg) (77). The ligands were docked at each receptor using a grid box of 36 Å^3^ centered in the catalytic pocket. The grid center for both proteins was X = 25.244 Å, Y = 12.841 Å, and Z = 7.184 Å. Docking results were computed in Autodock Vina (78) based on an exhaustiveness of four hundred one mode. The docking controls were HMG-CoA (the enzyme substrate) and simvastatin (an inhibitor of HMGR).

### Toxicity of the supernatant in the *Galleria mellonella* model

The toxicity test began by injecting an initial dose of the supernatant (5 mg/kg body weight) into five larvae. Larva mortality was recorded daily for five days. If three or more larvae died, the compound was assigned to the highest class of toxicity (GHS 1). If three or more larva survived, the initial dose was retested on a new group of larvae. If three or more larvae were again found to survive, a higher dose (25 mg/kg body weight) was tested. The experiment continued until a toxic dose was established. If the compound was not toxic at the highest dose (2,000 mg/kg), the compound was established as nontoxic (79). Isotonic saline solution was used as the control of nontoxicity and DMSO as the control of toxicity.

## Data Availability

The *S. albidoflavus* Q genome sequence generated was deposited in GenBank under BioProject number PRJNA886754 and annotation number GCA_025630915.1.

## SUPPLEMENTAL MATERIAL

Supplemental material spectrum.01271-23-s0001.pdf Table S1, Fig. S1, and Fig. S2.

ASM does not own the copyrights to Supplemental Material that may be linked to, or accessed through, an article. The authors have granted ASM a non-exclusive, world-wide license to publish the Supplemental Material files. Please contact the corresponding author directly for reuse.
