# Supplementary material for: Streptomyces albidoflavus Q antifungal metabolites inhibit the ergosterol biosynthesis pathway and yeast growth in fluconazole-resistant Candida glabrata: phylogenomic and metabolomic analyses
Source: Microbiol Spectr. 2023 Sep 27;11(5):e01271-23. doi: 10.1128/spectrum.01271-23 (PMC10581079; doi:10.1128/spectrum.01271-23)
Supplement: Supplemental material — Table S1, Fig. S1, and Fig. S2. [file spectrum.01271-23-s0001.pdf]

SUPPLEMENTAL MATERIAL

Bautista-Crescencio *et al.*

Suppl. TABLE 1. Effect of lyophilized supernatant of *S. albidoflavus* strain Q on the growth of *Candida* spp.

	**Inhibition (% of relative growth ± SD)**			
***Streptomyces* Q lyophilized supernatant concentration (µg/mL)**	***C. albicans* ATCC 10231**	***C. krusei* ATCC 14423**	***C. glabrata* CBS 138**	***C. glabrata* CGL 43**
**0**	0	0	0	0
**4.8**	23.17 ± 3.44	9.39 ± 7.56	75.66 ± 5.35	14.22 ± 7.15
**9.7**	61.77 ± 2.26	7.98 ± 0.81	85.27 ± 0.71	24.15 ± 2.86
**19.5**	92.05 ± 1.47	37.56 ± 4.23	88.53 ± 8.98	69.93 ± 0.68
**39**	99.08 ± 1.21	83.10 ± 11.38	95.81 ± 0.93	88.89 ±1.94
**78**	100. ± 0.00	99.53 ± 0.47	100 ± 0	95.41 ± 0.26
**156**	99.69 ± 0.27	99.69 ± 0.27	100 ± 0	100.00 ± 0
**312**	99.85 ± 0.27	99.69 ± 0.27	100 ± 0	99.85 ± 0.26
**625**	99.54 ±0.00	99.37 ± 0.27	100 ± 0	99.85 ± 0.26
**1250**	99.69 ± 0.27	99.53 ± 0.47	99.85 ± 0.27	99.85 ± 0.26
**2500**	99.54 ±0.00	98.75 ± 0.72	99.85 ± 0.27	99.85 ± 0.26
**5000**	99.24 ± 0.53	98.90 ± 0.72	99.69 ± 0.54	99.26 ± 0.51
**10000**	100 ± 0	100 ± 0	100 ± 0	100 ± 0

The relative growth was calculated as a percentage of the growth detected in the absence of any inhibitor, considered 100%. The original results were obtained by optical density, determined in a Thermo Scientific Multiskan FC microplate photometer at 620 nm (OD_{620}), after incubation for 24 h at 37°C. The quantification of growth was based on OD values which were expressed as the average of three independent essays ± SD.

Suppl. TABLE 2. Toxicity test of the supernatant of *S. albidoflavus* Q.

**Solution**	**LD_{50} (mg/kg body weight)**
**Saline solution**	> 2000
**DMSO**	125
**Supernatant of *S. albidoflavus* Q**	> 2000

The possible toxicity of the metabolites found in the supernatant of *S. albidoflavus* Q was evaluated by using the *Galleria mellonella* model. No toxicity was observed, as shown by the 50% dimethyl sulfoxide (DMSO) toxicity control.

Suppl FIG. 1 Metabolites found in metabolomic assays of *S. albidoflavus* strain Q.

Stigmatellin Y

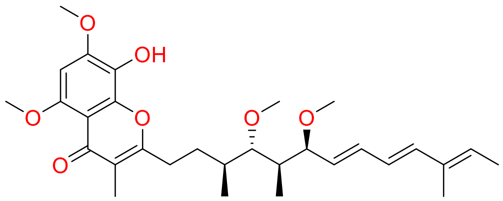

3'-Hydroxy-4'-methoxyglabridin

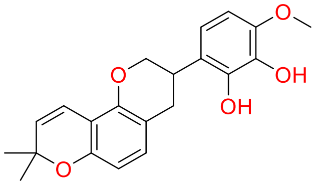

Aminopropylcadaverine

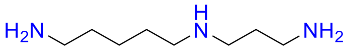

L-Agaridoxin

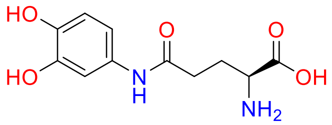

Notoginsenoside J

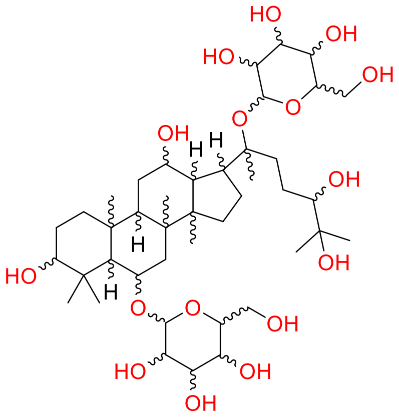

Isonicotinamide (Isonicotineamide)

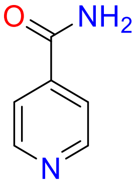

Azacitidine

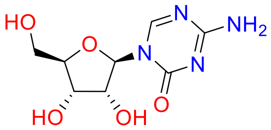

Nitrilacarb

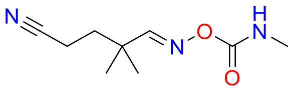

[1-(Hydroxymethyl)-5,5-dimethyl-2,4-imidazolidinedione]

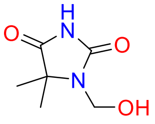

Dihydrocaffeic acid 3-O-glucuronide

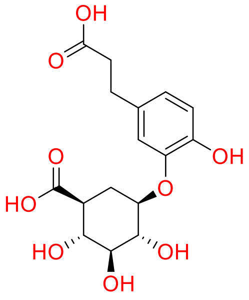

4-Methylaminobutyrate

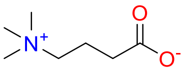

D-Fuconate

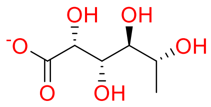

3-Methoxy-4,5-methylenedioxybenzoic acid

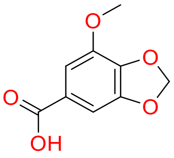

N6,N6-Dimethyladenosine

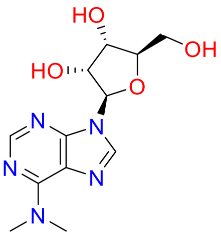

1-Nitrohexane

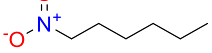

Tovophyllin B

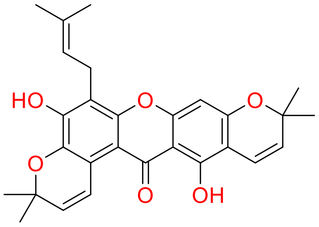

5-Methoxypyrrolidin-2-one (Pterolactam)

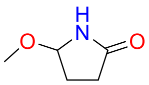

Isoguanine (2-Hydroxyadenine)

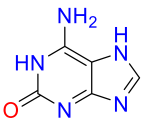

Benzyl 2-aminoacetate (Benzyl glycinate)

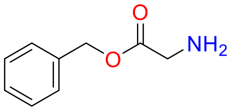

Azacitidine

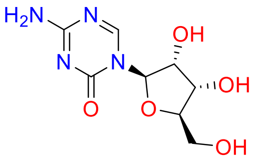

4-(3-Pyridyl)-3-butenoic acid

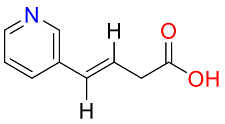

Sarracine

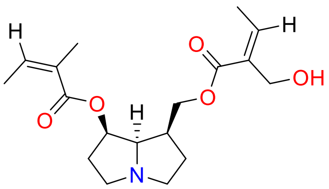

Pantothenic Acid

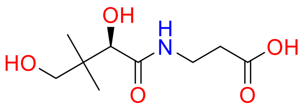

Tranexamic acid

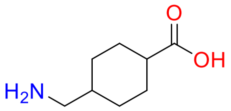

Indanofan

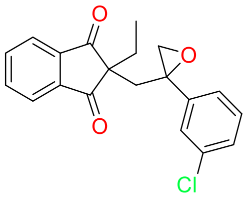

4-Hydroxy-3,4-dihydro-2H-pyrrole-2-carboxylic acid (Pyrroline hydroxycarboxylic acid)

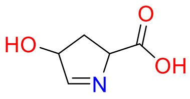

Isonicotinic acid

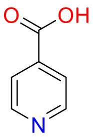

1,6-Dimethoxypyrene

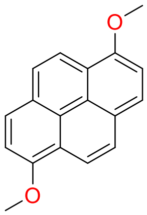

Methyl nicotinate

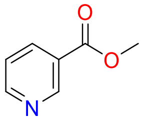

Epoxyeremopetasinorol

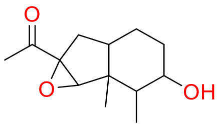

2-Propionyl-1-pyrroline (3,4-Dihydro-5-propanoyl-2H-pyrrole)

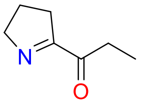

Hydroxyprolyl-Valine

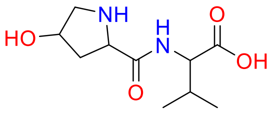

Sarmentosin

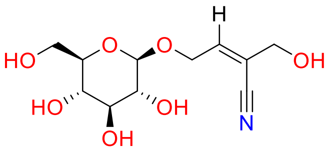

N-Acetylserine

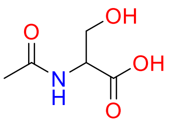

(S)-3-Hydroxyphenylglycine [3-Hydroxyphenylglycine]

Prolylserine (Prolyl-Serine)

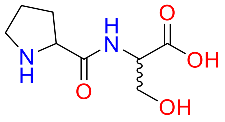

H-Thr-pro-OH (Threoninyl-Proline)

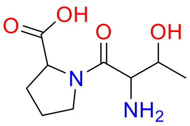

N2-Acetyl-L-aminoadipate

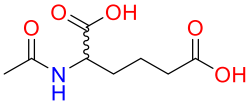

Hydroxyprolyl-Hydroxyproline

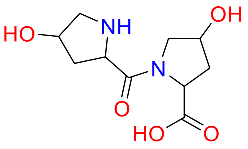

Asparaginyl-Proline

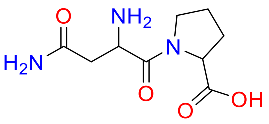

Pymetrozine

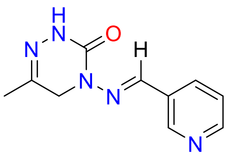

4-Hydroxyproline galactoside

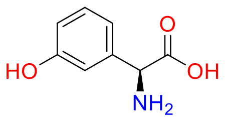

Aspartyl-Leucine

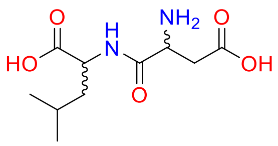

L-beta-Aspartyl-L-leucine

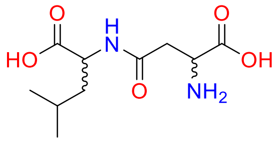

(3r,11Bs)-9-methoxy-1,3,4,6,7,11b-hexahydro-2h-pyrido[2,1-a]isoquinoline-3-carboxylic acid [Cis-1,3,4,6,7,11b-Hexahydro-9-methoxy-2H-benzo[a]quinolizine-3-carboxylic acid]

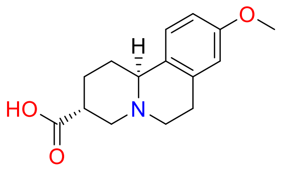

L-Saccharopine (Saccharopine)

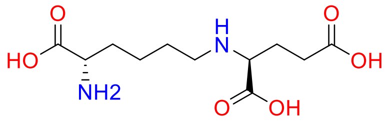

Formylisoglutamine

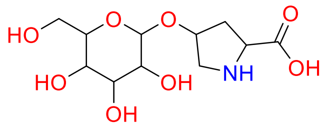

Threoninyl-Hydroxyproline

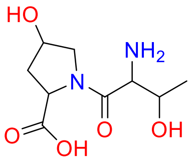

Beta-Ionone epoxide

[(5R,6S)-5,6-Epoxy-7-megastigmen-9-one]

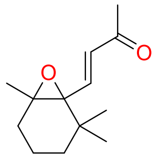

2-(3-Phenylpropyl)tetrahydrofuran

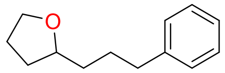

Iminodiacetic acid (Diglykokoll)

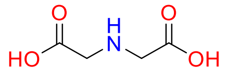

AminoDHQ

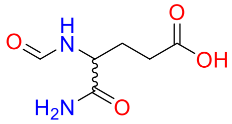

Dioscoretine

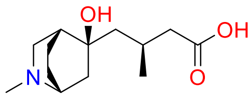

Gamma-Glutamyltyrosine

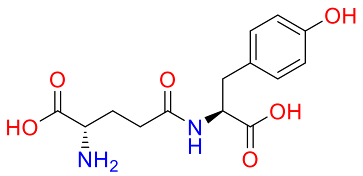

2,7-Anhydro-alpha-N-acetylneuraminic acid

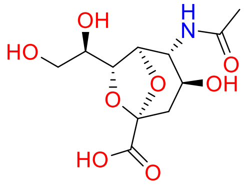

9-O-Acetylneuraminic acid

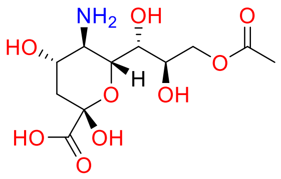

Isogingerenone B

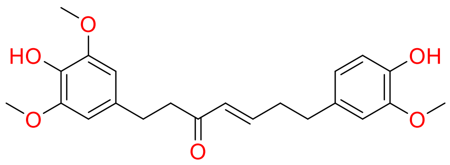

Armillarin

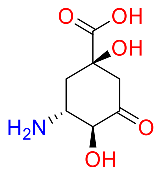

2(α-D-Mannosyl)-D-glycerate

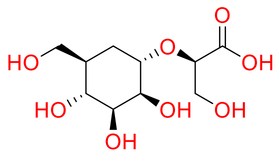

11-Amino-undecanoic acid

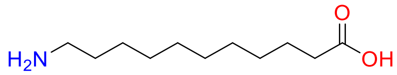

12-Ketodeoxycholic acid

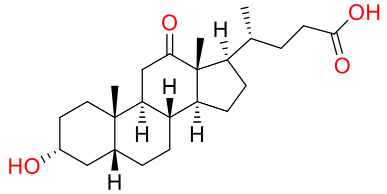

Polyoxyethylene (600) mono-ricinoleate

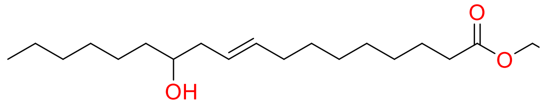

1-Stearoyl-sn-glycerol [MG(18:0/0:0/0:0)]**

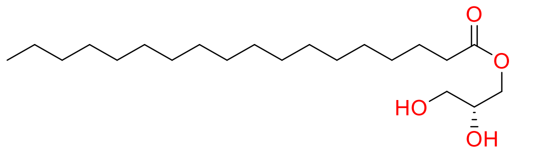

Hexadecasphinganine

(C16 Sphinganine)

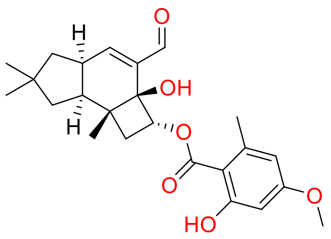

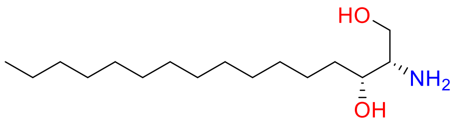

Suppl Fig. 2 Validation of the HMGR enzyme model of *C. glabrata* (Ramanchandran plot).

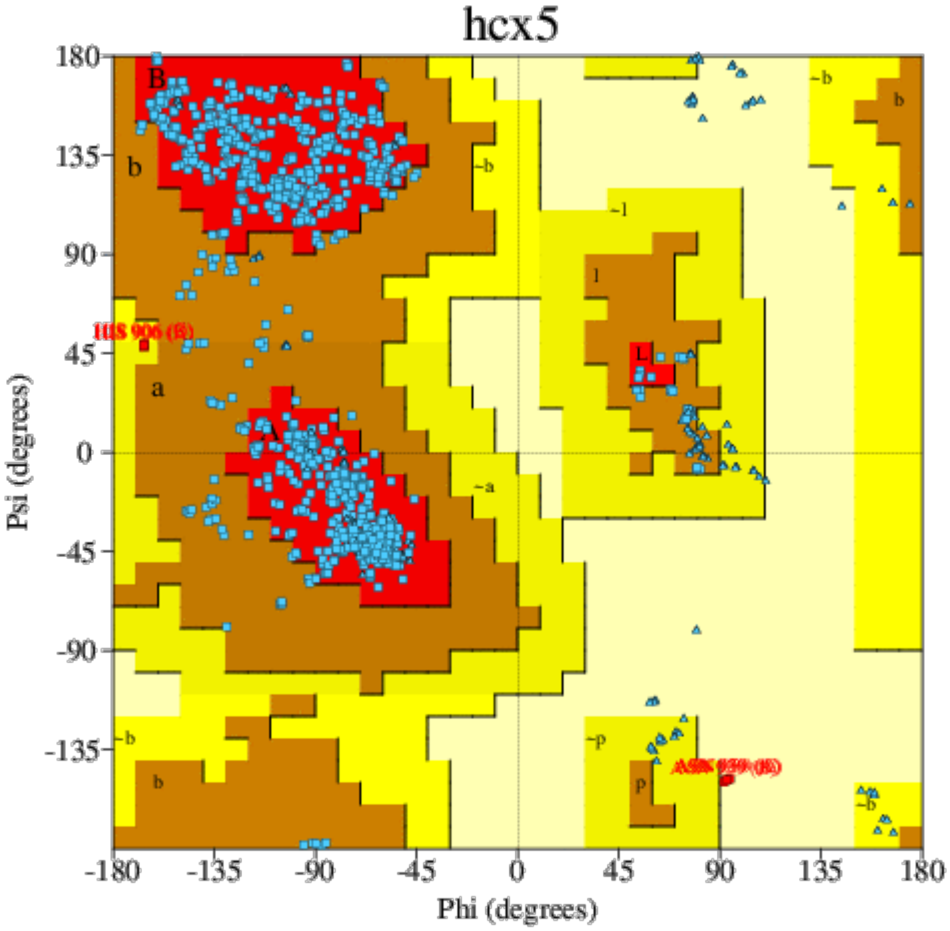

	No. of residues	%-tage
	------	------
Most favoured regions [A,B,L]	1288	91.2%
Additional allowed regions [a,b,l,p]	117	8.3%
Generously allowed regions [~a,~b,~l,~p]	4	0.3%
Disallowed regions [XX]	4	0.3%*
	----	------
Non-glycine and non-proline residues	1413	100.0%

End-residues (excl. Gly and Pro)	7
Glycine residues	167
Proline residues	71

Total number of residues	1658
